# Colorimetric glucose biosensing based on peroxidase-mimicking activity of Fe,N co-doped carbon dots

**DOI:** 10.1039/d6ra01116k

**Published:** 2026-07-02

**Authors:** Ola G. Hussein, Noreen Mohamed, Noha I. Abdelaziz, Amr M. Mahmoud, Kholoud Ahmed

**Affiliations:** a Department of Pharmaceutical Chemistry, Faculty of Pharmacy, Future University in Egypt Cairo 11835 Egypt ola.farag@fue.edu.eg; b Department of Chemistry, School of Pharmacy, Newgiza University Km. 22 Cairo-Alex Road Giza P.O. Box 12577 Egypt; c Department of Pharmaceutical Analytical Chemistry, Faculty of Pharmacy - Cairo University Kasr El-Aini Street ET-11562 Cairo Egypt amr.bekhet@pharma.cu.edu.eg; d Pharmaceutical Analytical Chemistry Department, Faculty of Pharmacy, Fayoum University Fayoum 63514 Egypt

## Abstract

For over two centuries, natural enzymes have played essential roles in biochemistry and have found diverse applications in medicine, healthcare, food production, and the chemical industry. However, natural enzymes often face limitations such as high cost, limited stability, and challenging storage conditions. Artificial enzymes offer significant advantages including cost-effective and controllable synthesis, tunable catalytic properties, and enhanced stability under harsh conditions. In this work, iron and nitrogen co-doped carbon dots (Fe,N-CDs) were synthesized *via* a simple, rapid, and cost-effective pyrolysis method using glutamic acid as the carbon source, allantoin as the nitrogen source, and ferric chloride as the iron source. The synthesized Fe,N-CDs were characterized using scanning electron microscopy (SEM), high-resolution transmission electron microscopy (HRTEM), energy-dispersive X-ray spectroscopy (EDX), Fourier-transform infrared (FT-IR) spectroscopy, and fluorescence spectroscopy confirming successful doping and revealing excitation at 235.9 nm and emission at 325.0 nm. The Fe,N-CDs exhibited remarkable peroxidase-like activity catalyzing the oxidation of peroxidase substrates in the presence of hydrogen peroxide (H_2_O_2_). A linear response was observed for hydrogen peroxide in the range of 25–600 µM, while glucose detection coupled with glucose oxidase (Gox) showed proportional absorption changes in the range of 50–600 µM enabling indirect quantification of glucose. These results demonstrate that Fe,N-CDs provide a simple, robust, and cost-effective platform for colorimetric biosensing highlighting their potential applications in medical diagnostics, clinical evaluation, and home healthcare monitoring.

## Abstract 2

Fe,N-doped carbon dots as nanozymes for sensitive colorimetric glucose detection. Glucose oxidase generated H_2_O_2_ from glucose. Fe,N-CDs catalyze OPD oxidation by H_2_O_2_ and generate a yellow color signal proportional to glucose concentration.

## Graphical abstract

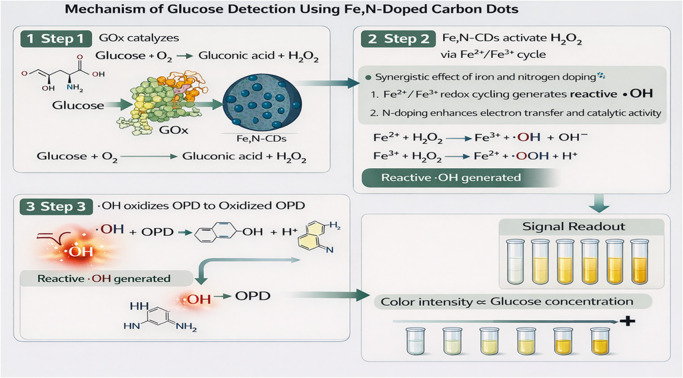

## 1. Introduction

Over many decades, extensive research has been dedicated to natural enzymes which are vital to biochemistry.^1^ Beyond their essential roles these enzymes have diverse applications in fields such as medicine, healthcare, food production, and the chemical industry.^2,3^ They play critical roles in various biological processes including metabolism, DNA replication, and cellular signaling. Their remarkable characteristics such as efficient catalysis under mild conditions and high substrate specificity have established them as invaluable resources. However, because most natural enzymes are proteins they are vulnerable to denaturation from environmental changes, degradation by proteases, and fluctuations in pH and temperature.^4,5^ Furthermore, challenges like high acquisition costs and storage issues can significantly affect their activity and usability.

Artificial enzymes have garnered significant interest because of their benefits compared to natural enzymes including cost-effective and controlled synthesis, tunable catalytic properties, and enhanced stability under harsh conditions.^6–21^ Various nanomaterials have demonstrated remarkable enzyme-like activities leading to significant advancements in this field because of the distinctive properties of nanomaterials. Nanozymes, which are nanomaterial-based artificial enzymes have been applied in various fields in areas such as biosensing, cancer therapy, energy transfer, and pollution remediation.^22^ Among these, peroxidase-like nanozymes have garnered significant attention because of their close association with oxygen reduction reactions (ORR) and their broad relevance across multiple disciplines. Nanomaterials, including metal nanoparticles, carbon-based nanomaterials, and other nanocomposites exhibit peroxidase-like activity to varying degrees. However, current catalytic materials still fall short of fulfilling the combined requirements of high catalytic efficiency, long-term durability, and affordability for practical applications.^23^ Thus, the development of a durable and efficient nanomaterial that can serve as a reliable peroxidase mimic is highly sought after.^24^

Quantum dots are nanoscale semiconductor crystals that typically range from 2 to 10 nm in size.^25,26^ They possess remarkable optical properties including size-tunable photoluminescence which allows for precise control over the emitted light spectrum. These nanocrystals have a high extinction coefficient and a significant fluorescent quantum yield making them highly effective in various applications.^27^ They also demonstrate excellent stability against photo-bleaching. Additionally, their characteristic fluorescence intermittency or blinking behavior introduces a dynamic quality that enhances their potential for advanced research and innovative applications.^28–33^

Due to their significance technological and fundamental nanomaterials particularly functional nanomaterials have garnered significant interest in recent years.^34,35^ Their large surface to volume ratio makes them attractive for use as high-efficiency catalysts leading to extensive research over the past decade.^36^ Both heterogeneous and homogeneous catalysis can be achieved *via* transition metal nanomaterials.

Recent studies have shown that nitrogen-doped carbon dots (N-CDs)^37–39^ effectively address the limitations of pure carbon dots (CDs) by introducing a single energy level leading to expanded applications in fields like photocatalysis, biosensing, energy storage, and pollutant degradation.^40–45^ In recent years, significant efforts have focused on doping carbon dots with various heteroatoms to enhance their properties. Iron, in particular has shown broader catalytic activity in addition to ferriporphyrin including proteins also in various iron carbide nanoparticles with its catalytic performance often attributed to the binding of iron among nitrogen in carbon-based structures. Despite these advances, complex preparation methods and limited modification sites have hindered the large-scale production and further applications of iron-doped CDs. N-CDs offer a promising alternative typically synthesized *via* simple pyrolysis of carbon and nitrogen-containing materials. By incorporating iron into the N-CD structure it is possible to achieve excellent catalytic properties.^39^ This motivates the development of an efficient straightforward method for synthesizing iron-doped carbon dots with enhanced catalytic performance aimed at expanding their practical applications.^46^

Allantoin and glutamic acid serve as effective nitrogen and carbon source for quantum dots enhancing their stability, solubility, and biocompatibility while also influencing their optical properties.^47,48^ By incorporating these compounds into the synthesis and functionalization of quantum dots their performance can be significantly improved for applications in areas such as biosensing, imaging, drug delivery, and environmental monitoring. Furthermore, the use of pyrolysis in combination with allantoin and glutamic acid sets this approach apart as it has not been explored in previous studies on iron and nitrogen co-doped carbon dots. While several papers have been published on this topic none have utilized pyrolysis along with allantoin and glutamic acid making this approach particularly novel and promising.^49,50^

In this research, iron and nitrogen co-doped carbon dots (Fe,N-CDs) were synthesized using a one-pot rapid pyrolysis technique. The resulting Fe,N-CDs displayed a uniform shape with exceptional water dispersibility. Due to incorporation of iron these particles exhibited intrinsic peroxidase mimicking activity Fe,N-CDs demonstrated superior catalytic stability across an acidic pH range and fluctuating temperature conditions greatly expanding their potential purposes. A colorimetric approach for hydrogen peroxide and glucose detection was established using Fe,N-CDs as catalyst. Results obtained highlight the promising potential of Fe,N-CDs in biosensing and medical diagnostics.

## 2. Experimental

### 2.1. Instruments and software

Scanning electron microscopy (SEM), (The Quanta FEG-250), high-resolution transmission electron microscope (HRTEM), JEM 2100 HRT, Japan), energy-dispersive X-ray spectroscopy (EDX) were applied to measure and characterize the various morphologies of the synthesized nanoparticles. The analysis of chemical bonds and detection of functional groups was carried out by Fourier-transform infrared (FT-IR) spectroscopy with a Shimadzu IR 435 spectrometer.

Spectrophotometric measurements were carried out using a SHIMADZU UV-vis spectrophotometer dual-beam Model UV-1800 PC (Kyoto, Japan) with UV Probe software version 2.43. Measurements were performed using the instrument's standard settings with a spectral bandwidth of 1 nm and a wavelength scanning speed of 2800 nm/min and 1 cm quartz cells were used.

### 2.2. Materials and reagents

Double-distilled water was used throughout the work obtained from Ultra-pure water system – ELGA PURELAB flex (PF3XXXXM1) (UK). *O*-phenylenediamine (OPD), acetic acid-sodium acetate buffer pH 4.0 and ammonia were acquired from Piochem (Cairo, Egypt). Glutamic acid, allantoin, ferric chloride, and hydrogen peroxide were purchased from Sigma Aldrich (Germany).

### 2.3. Iron and nitrogen co-doped carbon quantum dots synthesis

A facile one-step pyrolysis synthesis method was employed for the preparation of Fe,N-CDs. 1.0 g of glutamic acid a readily available biocompatible amino acid was combined with 1.0 g of allantoin a purine derivative known for its chelating properties and ferric chloride as the iron source. This dry powder mixture was then placed in a porcelain dish on hot plate and heated at 270 °C for 50 minutes. After pyrolysis process, the mixture was allowed to cool to ambient temperature. The resulting product was then dispersed in 50 mL of distilled water to facilitate extraction and purification. The dispersed mixture was then centrifuged for 30 minutes to remove any larger aggregates. Finally, the supernatant containing the synthesized Fe,N-CDs was filtered through a 22 µm syringe filter to obtain a monodispersed solution. The purified Fe,N-CDs were stored at 4 °C for further characterization and application.

### 2.4. Characterization of iron doped quantum dots

The synthesized Fe,N-CDs were characterized using a combination of scanning electron microscopy (SEM), high-resolution transmission electron microscopy (HRTEM), energy-dispersive X-ray spectroscopy (EDX), fluorescence measurements, and fourier-transform infrared spectroscopy (FT-IR). SEM analysis was performed to provide detailed insights into the morphology, size, and shape of the Fe,N-CDs. HRTEM analysis was performed to investigate the nanoscale structural features and to determine particle size and crystallinity. EDX was employed to analyze Fe,N-CDs elemental composition generating elemental maps to visualize the distribution of elements and offering quantitative data on the atomic percentages of each constituent element. This characterization was crucial for understanding the material's essential properties and potential uses. Fourier-transform infrared spectroscopy (FT-IR) was carried out in the range of 4000–400 cm^−1^ to identify functional groups within Fe,N-CDs.

Additionally, to morphological and compositional analysis, Fe,N-CDs fluorescence properties were investigated employing a specialized fluorimetric instrument. This enabled precise measurement of the fluorescence emission spectra providing insights into the energy levels and radiative transitions within the nanoparticles.

### 2.5. Hydrogen peroxide detection using Fe,N-CDs as peroxidase mimetics

To examine the synthesized Fe,N-CDs peroxidase-like activity, the peroxidase substrate OPD catalytic oxidation in the presence of H_2_O_2_ was assessed using UV-vis spectrophotometry method. First, 100 µL of 60 mM OPD, 600 µL of 100 mM H_2_O_2_ and 100 µL of the Fe,N-CDs stock solution were added together to 900 µL of 0.2 M acetate buffer (pH 4.0) in a quartz cuvette. Then in a 40 °C water bath the resulting solution was incubated for 10 minutes. Afterwards, the UV-vis spectra for the solution were recorded over a scanning range of 300.0–700.0 nm and the absorbance of OPD was measured at 450.0 nm to evaluate the reaction's progress. Finally, control experiments were conducted by repeating the same procedures but without the use of the hydrogen peroxide.

### 2.6. Influence of buffer pH on catalytic activity

To investigate the effect of buffer pH reaction going on Fe,N-CDs, 0.2 M acetate buffer solutions with pH values ranging from 3.0 to 6.0 were prepared and analyzed under the same experimental conditions as previous mentioned under Section 2.5.

### 2.7. Influence of temperature incubation

To assess the impact of incubation temperature on the activity of Fe-N-CDs, the temperature varied within a range of 20 to 80 °C using water baths. The absorbance of the resulting yellow solutions was subsequently monitored using UV-vis spectrophotometry across the different tested pH values and temperatures. This allowed for the determination of the optimal conditions for Fe,N-CDs catalytic activity.

### 2.8. Influence of incubation time

In addition, the effect of incubation time on the performance of the sensing method was systematically studied by varying the reaction duration between (10–15–20–25 min). This assessment aimed to determine the optimal time required to achieve a stable and measurable signal ensuring both accuracy and efficiency in glucose detection.

### 2.9. Calibration curve construction for hydrogen peroxide

For the calibration of the samples, a calibration curve was constructed using a range of standard solutions with varying concentrations of hydrogen peroxide stock solution (10 to 600 µM) using the same procedure and quantities as in the previous mentioned under section 2.5, followed by measuring the absorbance at the maximum wavelength of 450.0 nm.

### 2.10. Glucose detection using GOx and Fe,N-co-Doped carbon quantum dots

Glucose detection was performed as follows: 20 µL of 20 mg/mL glucose oxidase and 24 µL of glucose at varying concentrations in acetate buffer 50 mM (pH 5.1) were incubated for 30 minutes at 37 °C. Then, 24 µL of 60 mM OPD, 10 µL of the Fe,N-CDs stock solution, and 24 µL of 100 mM H_2_O_2_ were added to 185 µL of acetate buffer 0.2 M at pH 4.0, and then mixture undergoes incubation for 15 minutes in a 40 °C water bath.

### 2.11. Kinetic analysis of Fe,N-CDs peroxidase-like activity

To evaluate the catalytic efficiency of the synthesized Fe,N-CDs, steady-state kinetics were performed using hydrogen peroxide as the substrate. Michaelis–Menten parameters (*K*_m_ and *V*_max_) were determined from Lineweaver–Burk plots to assess substrate affinity and maximum catalytic rate. For comparison, representative peroxidase-mimicking nanozymes reported in the literature were included such as ZnFe_2_O_4_/GQDs nanocomposites^51^ and N-CDs/Fe_3_O_4_ magnetic nanozymes.^52^

### 2.12. Selectivity evaluation of the Fe,N-CDs-based glucose sensing system

The selectivity of the Fe,N co-doped carbon dots (Fe,N-CDs) as peroxidase mimetics for glucose detection was systematically investigated through interference studies conducted under optimized experimental conditions. To assess the specificity of the sensing platform, a range of potentially coexisting substances commonly found in biological samples were examined including ascorbic acid, uric acid, fructose, lactose, sucrose, and citric acid.

## 3. Result and discussion

### 3.1. Structural and compositional analysis of materials

A comprehensive characterization was carried out to investigate the morphological, structural, compositional, and optical properties of the synthesized Fe,N-CDs. Multiple analytical techniques were employed to confirm successful formation and functional characteristics of the prepared nanomaterial.

**Fig. 1:**
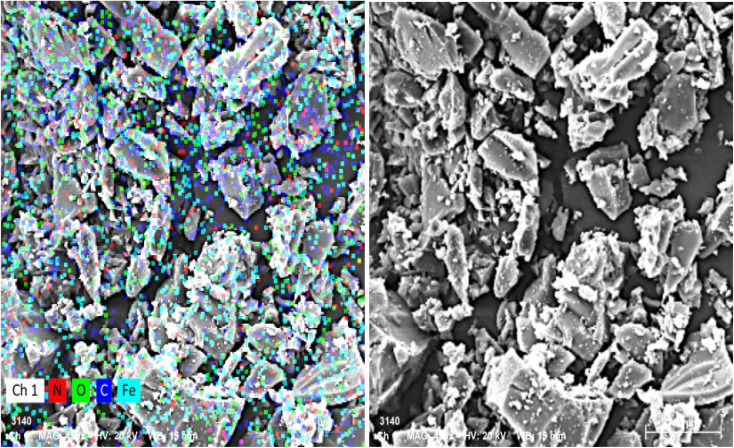
The SEM images of Fe,N-CDs EDS and elemental mapping in Fe,N-CDs.

The nature of surface morphology and distribution were visualized by SEM micrographs. As shown in Fig. 1, the SEM images of the prepared Fe,N-CDs reveal particles with small lamellar and square-shaped sheet-like structures indicating the formation of carbon-based nanostructured materials.

**Fig. 2:**
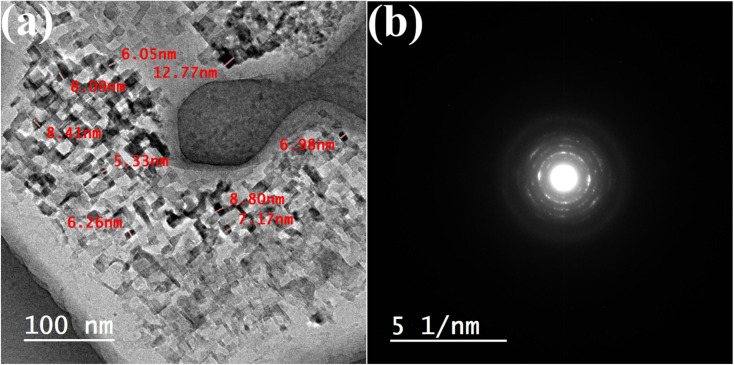
(a) TEM image showing nanoscale domains with measured particle sizes, and (b) corresponding SAED pattern displaying concentric diffraction rings indicating polycrystalline structure.

To further investigate the structural characteristics at the nanoscale, high-resolution transmission electron microscopy (HRTEM) analysis was performed as shown in Fig. 2. The HRTEM images clearly reveal uniformly distributed nanoscale domains with sizes ranging from approximately 5 to 13 nm. Distinct lattice fringes are observed confirming the crystalline nature of these nanostructures; Fig. 2a. In addition, the corresponding selected area electron diffraction (SAED) pattern exhibits concentric diffraction rings indicating the presence of nanocrystalline domains; Fig. 2b. These results provide direct evidence for the formation of Fe–N–C quantum dot-like structures.

**Fig. 3:**
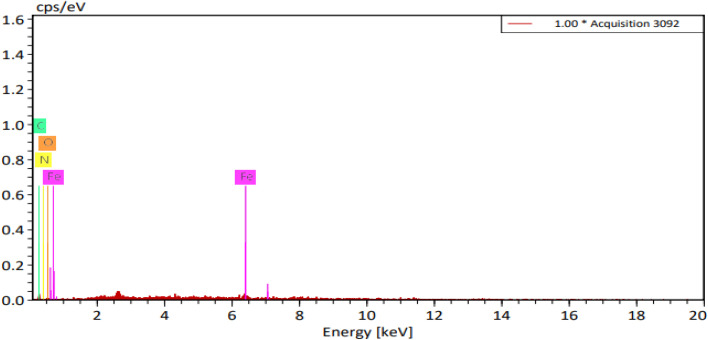
EDX spectrum of Fe,N-CDs showing the presence of Fe,C, N, and O elements.

**Fig. 4:**
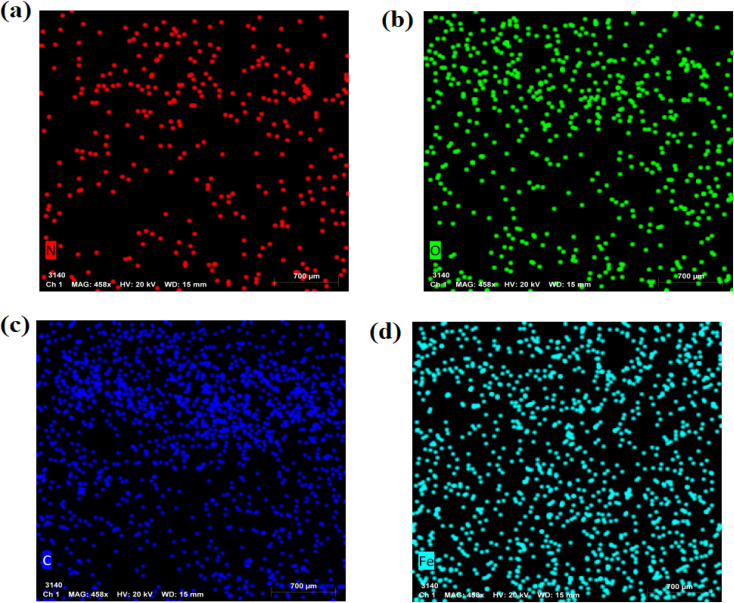
Elemental EDX mapping of Fe,N-CDs; (a) nitrogen, (b) oxygen, (c) carbon, and (d) iron.

Energy-dispersive X-ray spectroscopy (EDX) and elemental mapping were employed to analyze the elemental composition and spatial distribution of the synthesized material. The EDX spectrum reveals prominent peaks corresponding to iron (Fe), oxygen (O), nitrogen (N), and carbon (C) confirming the successful incorporation of these elements; Fig. 3. Elemental mapping further demonstrates their uniform distribution throughout the structure supporting the formation of a homogeneous Fe–N–C material; Fig. 4.

**Fig. 5:**
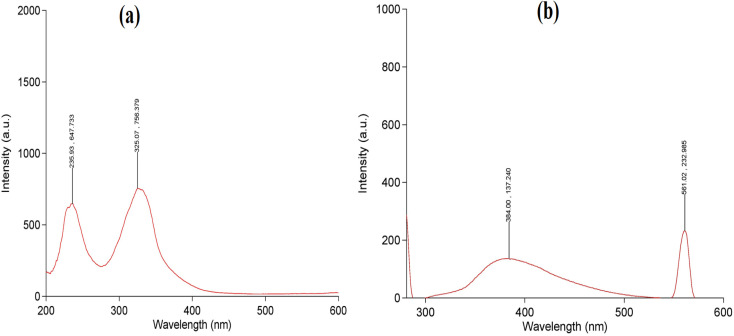
(a) Excitation and (b) emission spectra of the prepared Fe,N-CDs.

Fluorescence excitation and emission spectroscopy provided additional insight into the optical properties of the Fe,N-CDs. As shown in Fig. 5, the excitation spectrum exhibits a distinct peak at 235.93 nm with a corresponding emission peak at 325.07 nm. These optical characteristics are consistent with carbon-based quantum dot materials and confirm their successful synthesis. Furthermore, the obtained Fe,N-CDs demonstrate promising functional properties enabling their application as artificial enzymes for glucose detection.

The FTIR spectrum of Fe,N co-doped carbon quantum dots (Fe,N-CDs) confirms the presence of key surface functional groups. The broad band at 3200–3500 cm^−1^ corresponds to O–H/N–H stretching, while the peak near 2900 cm^−1^ is assigned to C–H vibrations. The band at 1700–1600 cm^−1^ indicates C=O/C=C groups and peaks at 1550–1400 cm^−1^ confirm C–N and N–H vibrations evidencing nitrogen doping. Additionally, bands in the 1200–1000 cm^−1^ range are attributed to C–O/C–N stretching. Weak signals at 600–500 cm^−1^ are associated with Fe–O/Fe–N bonds supporting iron incorporation; Fig. S1. Overall, these results verify the successful formation of Fe,N-CDs with functionalized surfaces.

Overall, the combined results from SEM, HRTEM, EDX, elemental mapping, fluorescence spectroscopy, and FTIR provide a comprehensive understanding of the synthesized Fe,N-CDs. The morphological and structural analyses confirm the formation of nanoscale crystalline quantum dot-like structures, while compositional studies verify the successful incorporation and uniform distribution of Fe, N, and C elements. Furthermore, the observed optical properties highlight their functional behavior supporting their suitability for catalytic applications. These findings collectively demonstrate the successful synthesis of Fe,N-CDs with desirable structural and functional characteristics making them promising candidates for use as artificial enzymes in glucose detection.

### 3.2. Peroxidase-mimicking activity of the assembled Fe,N-CDs

**Fig. 6:**
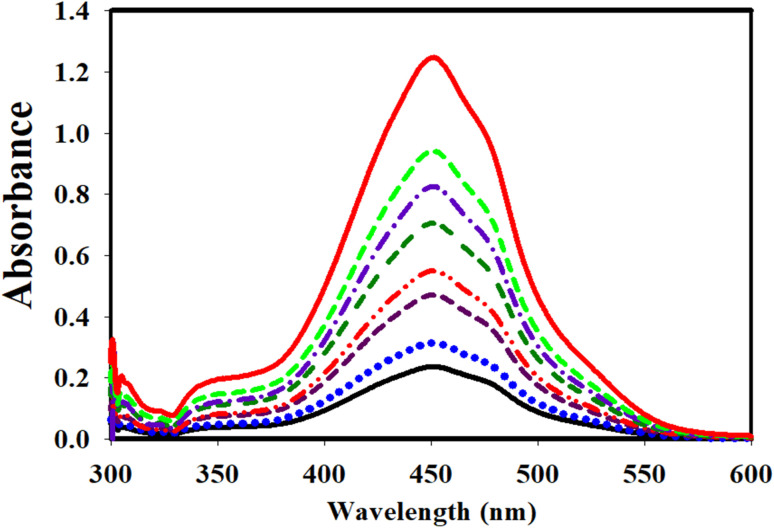
Overlaid UV-vis absorption spectra of the Fe,N-CDs catalytic system at varying hydrogen peroxide concentrations showing increased absorbance intensity with increasing hydrogen peroxide concentration.

The Fe,N-CDs peroxidase-mimicking activity were assessed through the peroxidase substrate OPD catalytic oxidation in the presence of hydrogen peroxide. Results indicated that the assembled Fe,N-CDs showed a catalytic performance in the OPD oxidation by hydrogen peroxide. The absorption spectra revealed an increased oxidation response highlighting the enhanced Fe,N-CDs catalytic performance towards OPD oxidation process mediated by hydrogen peroxide as shown in Fig. 6.

### 3.3. Optimization of Fe,N-CDs catalytic activity

#### 3.3.1. Influence of buffer pH on catalytic activity

**Fig. 7:**
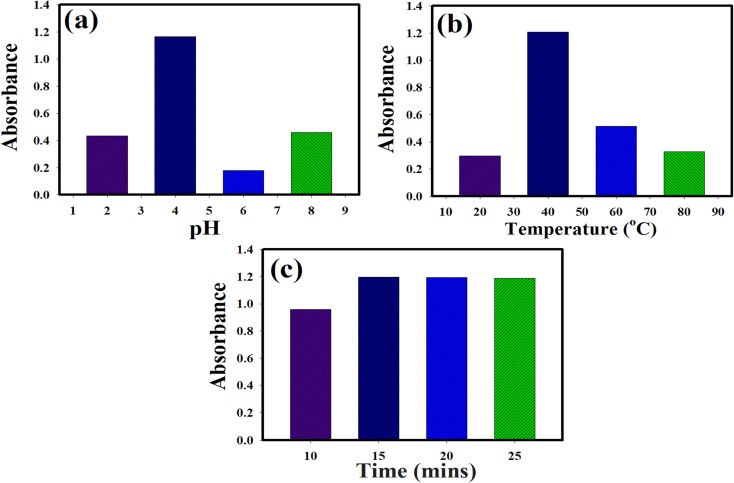
Influence of (a) pH, (b) temperature, and (c) time on catalytic activity of Fe,N-CDs.

The catalytic Fe,N-CDs activity is highly dependent on pH. Such study reveals that Fe,N-CDs catalyze the OPD oxidation with hydrogen peroxide more efficiently in acidic pH conditions with optimal performance observed at pH 4.0; Fig. 7a. Catalytic activity was detectable in buffer solutions with pH values above 4.0 while minimal activity was noted at pH values below 4.0. The highest absorbance values indicating the greatest catalytic efficiency were recorded at pH 4.0. Therefore, an acetate buffer 0.2 M at pH 4.0 was selected as such an optimal reaction medium for maximizing the Fe,N-CDs catalytic performance.

#### 3.3.2. Influence of temperature incubation

Furthermore, the temperature-dependent response curves for the reaction are presented in Fig. 7b. These curves demonstrate that the signals increased with rising reaction temperature. Notably, the highest absorbance was observed at 40 °C. This temperature was primarily chosen as it closely approximates physiological body temperature making the system more suitable for biological and practical applications. Therefore, 40 °C was determined to be the optimal reaction temperature for the current study.

#### 3.3.3. Influence of incubation time

Different time reaction intervals were studied (10–15–20–25 min) the response curves over time for the reaction are shown in Fig. 7c. These curves indicate that the signals increased as the reaction time was extended until it reached plateau. The highest absorbance was observed after 15 minutes. Therefore, a reaction time of 15 minutes was established as the optimal condition for the current study.

### 3.4. Optimization of Fe,N-CDs activity and application in hydrogen peroxide detection

The catalytic activity of the prepared Fe,N-CDs was systematically evaluated as a function of hydrogen peroxide concentration demonstrating a strong concentration-dependent response and confirming the suitability of the system for quantitative detection. Given the critical role of hydrogen peroxide in biomedical diagnostics and environmental monitoring, the development of reliable detection platforms is of significant importance. Hydrogen peroxide acts as a signaling molecule in various cellular processes and is closely associated with oxidative stress which is implicated in numerous diseases such as cancer, neurodegenerative disorders, and cardiovascular diseases. Therefore, accurate monitoring of hydrogen peroxide levels is essential for disease diagnosis and environmental analysis.

**Fig. 8:**
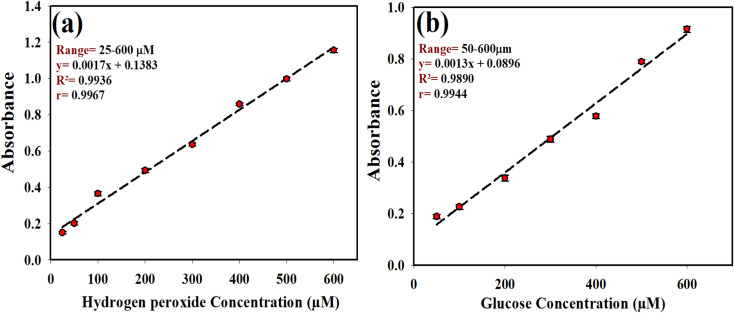
Calibration curves of the Fe,N-CDs catalytic system: (a) linear relationship between absorbance and hydrogen peroxide concentration (25–600 µM), and (b) linear response toward glucose concentration (50–600 µM) demonstrating high sensitivity and good correlation coefficients.

Under the optimized conditions (40 °C, 0.2 M acetate buffer at pH 4.0, and 15 min reaction time), the UV-vis absorption spectra (Fig. 6) show a gradual increase in absorbance intensity with increasing hydrogen peroxide concentration confirming the concentration-dependent catalytic activity of the Fe,N-CDs system. Based on these spectral changes, a calibration curve was constructed by plotting absorbance at the characteristic wavelength against hydrogen peroxide concentration; Fig. 8a. The calibration curve exhibited a linear range from 25 to 600 µM with a regression equation of *y* = 0.0017*x* + 0.1383. The high correlation coefficient (*R*^2^ = 0.9936, corresponding to *r* = 0.9967) indicates excellent linearity and reliability of the proposed sensing system. This confirms that the observed spectral changes can be reliably translated into a quantitative concentration-response relationship. The limit of quantification (LOD) was determined to be 23.52 µM.

The observed catalytic activity can be attributed to a Fenton-like mechanism as illustrated in Scheme 2 where Fe^3+^/Fe^2+^ redox cycling within the Fe,N-CDs facilitates the decomposition of hydrogen peroxide into highly reactive ˙OH radicals that drive the oxidation of OPD. In this process, iron centers act as active catalytic sites enabling efficient electron transfer, while nitrogen doping enhances the electronic structure promotes substrate adsorption and improves charge transfer efficiency thereby synergistically boosting the peroxidase-like activity.

To improve clarity and avoid redundancy, the key analytical parameters previously listed in Table 1 and 2 have been integrated into the graphical representation of the calibration curve. This integration provides a more concise and visually intuitive presentation of the assay performance. The strong linear correlation further confirms the robustness and reproducibility of the Fe,N-CDs-based detection platform highlighting its potential as a cost-effective and sensitive tool for hydrogen peroxide analysis.

**Table 1:** Assay parameters and results of hydrogen peroxide detection using the prepared Fe,N-CDs as artificial enzymes

Parameter	Hydrogen peroxide
Range (µM)	25–600

**Linearity**	
Slope	0.0017
Intercept	0.1383
Correlation coefficient (r)	0.9967
LOD^a^	23.52 µM
Accuracy (mean ± SD)^b^	100.26 ± 0.39
Precision, (% RSD)^c^	
– Repeatability	0.361
– Intermediate precision	0.698

a Limit of detection was calculated using the following equations: LOD = 3.3 *σ*/*S*, where “*σ*” is the mean of standard deviation of intercept and “*S*” is the slope.

b The accuracy, average of three concentrations (30.0, 350.0, 550.0 µM).

c The intra-day & inter-day (*n* = 3) relative standard deviation of concentrations (25.0, 200.0 and 400.0 µM).

**Table 2:** Assay parameters and results of glucose detection using GOx and the prepared Fe,N-CDs

Parameter	Glucose
Range (µM)	50–600

**Linearity**	
Slope	0.0013
Intercept	0.0896
Correlation coefficient (r)	0.9940
LOD^a^	47.0 µM
Accuracy (mean ± SD)^b^	101.74 ± 0.24
Precision, (% RSD)^c^	
– Repeatability	0.411
– Intermediate precision	0.733

a Limit of detection was calculated using the following equations: LOD = 3.3 *σ*/S, where “*σ*” is the mean of standard deviation of intercept and “*S*” is the slope.

b The accuracy, average of three concentrations (30.0, 350.0, 550.0 µM).

c The intra-day & inter-day (*n* = 3) relative standard deviation of concentrations (50.0, 200.0 and 400.0 µM).

### 3.5. Application of Fe,N-CDs for GOx-Based glucose detection

**Scheme 1:**
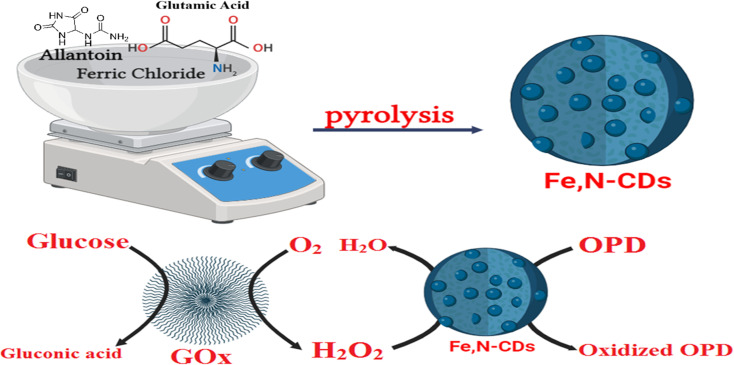
Schematic representation of Fe,N-CDs synthesis and colorimetric biosensing for glucose detection using GOx and the prepared Fe,N-CDs.

**Scheme 2:**
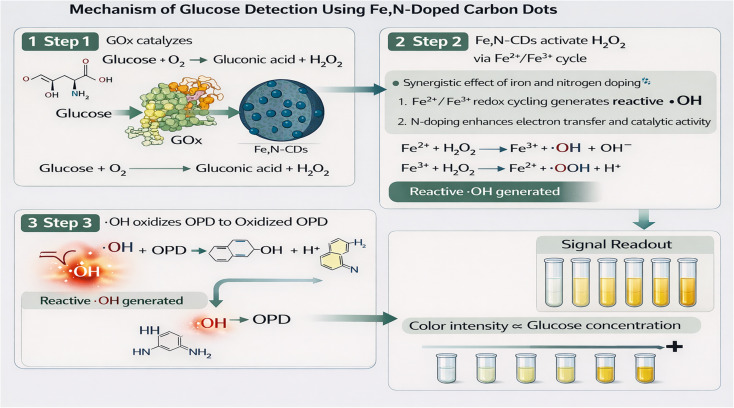
Schematic illustration of the Fenton-like peroxidase-mimicking mechanism of Fe,N-doped carbon dots for glucose detection *via* GOx-mediated hydrogen peroxide generation and ˙OH-driven OPD oxidation.

The detection of glucose is crucial for effective diabetes management and overall health monitoring. As glucose is the primary energy source for the body its levels have a direct impact on metabolic processes. Accurate and timely measurement of glucose is essential for individuals with diabetes to regulate insulin administration maintain blood sugar levels within a target range and prevent complications. Additionally, precise glucose detection is vital for the study of metabolic disorders contributing to the advancement of medical research, personalized medicine, and improved public health outcomes. In our study, we demonstrated that coupling the catalyzed oxidation of OPD by hydrogen peroxide using Fe,N-CDs as the catalyst with the reaction of glucose oxidation mediated through glucose oxidase enables effective colorimetric glucose detection; Scheme 1. Fig. 8b shows the glucose screening absorption profile applying the colorimetric developed method. The glucose detection process was carried out in two distinct and consecutive steps as outlined in the methodology due to the tendency of GOx to denature in neutral pH buffer solutions. The glucose oxidation reaction was carried out in a pH 5.1 buffer producing hydrogen peroxide. Subsequently, the hydrogen peroxide generated from the reaction of glucose oxidation was identified *via* the usage of Fe,N-CDs Scheme 1. As a result, our proposed colorimetric method offers a simple and effective approach for glucose detection. Fig. 8b presents a standard concentration-response curve of glucose demonstrating the ability to detect glucose concentrations as low as 50 µM with a linear range of detection from 50 to 600 µM; Table 2 and Scheme 2.

### 3.6. Comparative kinetic and analytical performance

The kinetic parameters and analytical characteristics of Fe,N-CDs and selected nanozyme systems are summarized in Table 3. The Fe,N-CDs exhibited a *K*_m_ of 0.045 mM indicating a higher affinity toward hydrogen peroxide than previously reported nanozymes. The *V*_max_ of 1.05 µM/s shows superior catalytic turnover compared to ZnFe_2_O_4_/GQDs nanocomposites^51^ and N-CDs/Fe_3_O_4_ magnetic nanozymes.^52^ The linear detection range of 25–600 µM and a limit of detection of 23.52 µM demonstrate that the Fe,N-CDs provide both high sensitivity and an extended working range for colorimetric glucose detection.

**Table 3:** Comparative kinetic and analytical performance of Fe,N-CDs and selected peroxidase-mimicking nanozymes for hydrogen peroxide detection

Nanozyme system	Substrate	*K*_m_ (mM)	*V*_max_ (µM/s)	Linearity (µM)	LOD (µM)	Reference
ZnFe_2_O_4_/GQDs nanocomposite	H_2_O_2_	0.068	0.083	5–500	7.0	51
N-CDs/Fe_3_O_4_ magnetic nanozyme	H_2_O_2_	0.607	0.205	1–180	0.56	52
Fe,N-CDs	H_2_O_2_	0.045	1.05	25–600	23.52	This work

### 3.7. Selectivity evaluation of the Fe,N-CDs-based glucose sensing system

The specificity of the Fe,N-CDs nanozyme-based glucose sensing platform was evaluated in the presence of common interfering species typically found in biological fluids. Compounds including citric acid, ascorbic acid, uric acid, lactose, fructose, and sucrose were individually tested at physiologically relevant concentrations to assess their influence on the detection signal. The results indicate that these substances produce negligible changes in absorbance compared to glucose under identical conditions confirming minimal interference; Table 4. The high specificity of the system is attributed to the selective oxidation of glucose-by-glucose oxidase and the efficient peroxidase-like catalytic activity of Fe,N-CDs toward the generated hydrogen peroxide. These findings demonstrate that the proposed sensing platform is reliable for accurate glucose determination in complex biological samples.

**Table 4:** Selectivity of the Fe,N-CDs system for glucose detection in the presence of interferents

Interferent	Concentration (µM)	Relative response (%)
Glucose	300	92.3
Ascorbic acid	300	2.5
Uric acid	300	3.8
Fructose	300	2.2
Lactose	300	1.9
Sucrose	300	5.6
Citric acid	300	3.3

## 4. Conclusion

In summary, the study successfully synthesized Fe,N-CDs which exhibited remarkable nanozyme-like peroxidase activity demonstrating their potential as efficient catalysts for hydrogen peroxide detection. Comprehensive characterization techniques including SEM, EDX, HRTEM, and fluorescence excitation-emission spectroscopy, FT-IR spectroscopy confirmed the structural, morphological, and compositional properties of the Fe,N-CDs. The catalytic oxidation of OPD in the presence of hydrogen peroxide resulted in a reliable colorimetric assay with a broad linear detection range from 25 to 600 µM for hydrogen peroxide. Moreover, the integration of glucose oxidase (GOx) with Fe,N-CDs enabled the development of a sensitive colorimetric method for glucose detection within a linear range of 50 to 600 µM. Importantly, the optimal catalytic performance was achieved under well-defined conditions, specifically at pH 4.0 using 0.2 M acetate buffer, a temperature of 40 °C, and an incubation time of 15 minutes. These parameters are critical for ensuring reproducibility and maximizing catalytic efficiency. The Fe,N-CDs demonstrated excellent stability across a broad range of temperatures and pH values, outperforming natural peroxidase enzymes in terms of robustness and resilience. These findings highlight the potential of Fe,N-CDs as effective and stable alternatives to natural peroxidases in various analytical applications particularly in biomedical diagnostics, research, and environmental monitoring. The performance demonstrated opens promising avenues for the development of cost-effective biosensing platforms for widespread applications in health monitoring and environmental protection.

## Author contributions

Ola G. Hussein, Noreen Mohamed, Noha I. Abdelaziz, Amr M. Mahmoud, and Kholoud Ahmed contributed equally to conceptualization, methodology, software, validation, formal analysis, investigation, resources, data curation, original draft preparation, visualization, supervision, and project administration, manuscript review and editing. All authors have read and agreed to the published version of the manuscript.

## Conflicts of interest

The authors declare no conflicts of interest.

## Supplementary Material

RA-OLF-D6RA01116K-s001

## Data Availability

All relevant data are included within the article. Supplementary information (SI) is available. See DOI: https://doi.org/10.1039/d6ra01116k.
