# Supplementary material for: Colorimetric glucose biosensing based on peroxidase-mimicking activity of Fe,N co-doped carbon dots
Source: RSC Adv. 2026 Jul 2;16(34):32189–201. doi: 10.1039/d6ra01116k (PMC13326240; doi:10.1039/d6ra01116k)
Supplement: RA-016-D6RA01116K-s001 [file RA-016-D6RA01116K-s001.pdf]

Supplementary Information (SI) for RSC Advances.
This journal is © The Royal Society of Chemistry 2026

Colorimetric Glucose Biosensing Based on Peroxidase-Mimicking Activity of Fe,N Co-Doped Carbon Dots

Ola G. Hussein[1*], Noreen Mohamed[2], Noha I. Abdelaziz[2], Amr M. Mahmoud[3*], Kholoud Ahmed[4]

[1]*Department of Pharmaceutical Chemistry, Faculty of Pharmacy, Future University in Egypt, Cairo, 11835, Egypt*

[2]*Department of Chemistry, School of Pharmacy, Newgiza University, Km. 22 Cairo-Alex Road, Giza P.O. Box 12577, Egypt*

[3]*Department of Pharmaceutical Analytical Chemistry, Faculty of Pharmacy - Cairo University, Kasr El-Aini Street, ET-11562, Cairo - Egypt*

[4]*Pharmaceutical Analytical Chemistry Department, Faculty of Pharmacy, Fayoum University, Fayoum, 63514, Egypt*

[*]Corresponding author's email: ola.farag@fue.edu.eg, amr.bekhet@pharma.cu.edu.eg

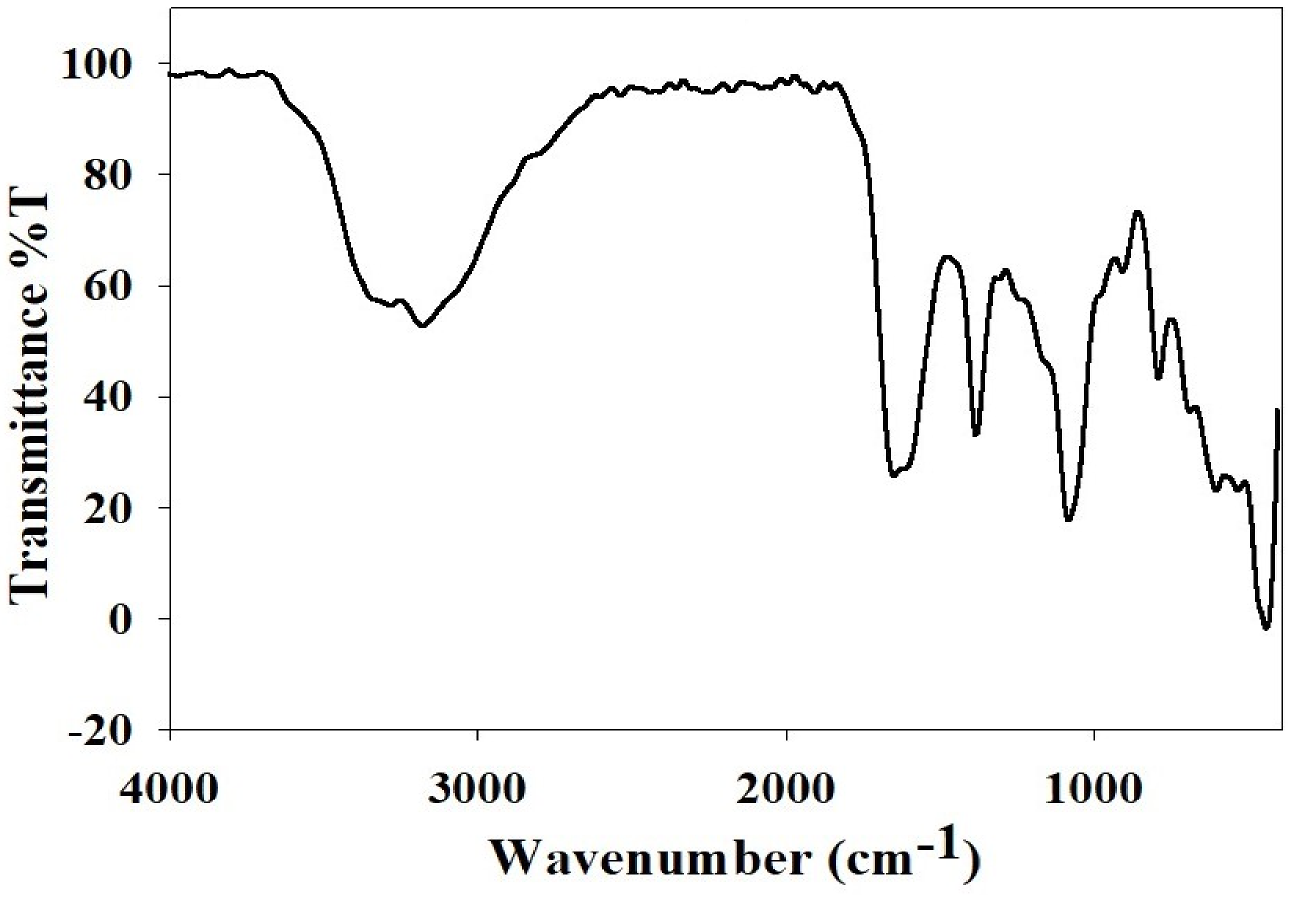

Fig. S1. FTIR spectrum of Fe,N co-doped carbon quantum dots (Fe,N-CDs) showing characteristic functional groups and evidence of Fe–N/Fe–O interactions.
